# Squamous cell carcinoma of the gallbladder

**DOI:** 10.1590/S1516-31802001000100010

**Published:** 2001-01-02

**Authors:** Jaques Waisberg, Sansom Henrique Bromberg, Maria Isete Fares Franco, Nagamassa Yamagushi, Paulo Amaral dos Santos, Mário Augusto Padulo Castro

Squamous cell carcinoma (SCC) of the gallbladder is a rare and aggressive affection and is responsible for up to 12.7% of the malignant neoplasms of this organ.^1,2,3^

It characteristically presents invasive growth, a low tendency towards lymph node metastases and a high incidence of local infiltration and hepatic metastases, presenting a worse prognosis than adenocarcinoma of the gallbladder.^1,2^

In the period between 1968 and 1998, three patients suffering from squamous cell carcinoma of the gallbladder were operated on in our Department. They died between 1 and 6 months after the surgery (Table 1).

**Table 1:** Principal aspects of the cases of squamous cell carcinoma (SCC) of the gallbladder

Case	Age	Sex	Finding	Operation	Lesion	Survival
1	68	female	invasion of hilum	Biopsy + T-tube	SCC grade III	1 month
2	52	female	hepatic metastasis	cholecystectomy	SCC grade II	6 months
3	74	female	invasion of hilum	biopsy	SCC grade III	2 months

SCC of the gallbladder is predominantly incident among females, in a proportion of 3:1 over males, and between the fourth and sixth decades of life,^1,2,3^ as was found in our cases.

Its rapid growth, early metastatic dissemination and diffuse local and regional infiltration characterize the biological behavior of the lesion. Such tumors tend to grow laterally along the *fossa* of the gallbladder, forming large infiltrative masses and invading the liver and adjacent organs (stomach, duodenum and trans-verse colon) by direct expansion.^1,2^ This pattern was verified in the cases here described. Despite this local and regional infiltration, it usually does not present metastases in lymph nodes, and seeding in the peritoneum is rare.^3^ Nevertheless, hepatic metastases, as seen in case 2, are more frequently found in SCC than in adenocarcinoma of the gallbladder.^1,2,3^

Most studies accept that the squamous cells originate from pre-existing metaplastic squamous epithelium; some others believe that SCC of the gallbladder originates from squamous differentiation of the adenocarcinoma cells, via expression of mixed phenotypes within a single tumor.^1,2,3^ Characteristically, the duplication time for SCC is half that of adenocarcinoma, such that the growth of SCC cells may overtake and substitute that of adenocarcinoma.^1,2^

Disease is suspected when the lesion reaches a large size and is locally advanced, which was also observed in the cases we studied.

The surgical options available depend mainly on the degree of local and regional involvement and consist of cholecystectomy with resection of a wedge of adjacent liver tissue or direct liver resection allied with regional lymphadenectomy and skeletization of the hepatic hilum.^2,4^

Resection of the organs involved as part of the radical operation is justified in cases of localized lesion, without metastases or peritoneal dissemination. Hepato-pancreatic duodenectomy was introduced as a radical treatment option for SCC of the gallbladder because of the type of dissemination seen in squamous cell carcinomas.^4^ However, its long-term benefits have not yet been satisfactorily documented.

Adjuvant postoperative radiotherapy and chemotherapy may be used, although their results are inconsistent and only palliative.^1,2^

The extent of the tumor at the time of diagnosis is the most important parameter in determining survival.^2,3^ The majority of the patients die around six months after diagnosis when radical surgery is not performed,^1,4^ as occurred with the patients described. These data reinforce the idea that early diagnosis is the most important parameter for improving the survival indices among patients with SCC of the gallbladder.
